# A deep dive into the surgical and pharmacological considerations of obesity in chronic myeloid leukemia: a narrative review

**DOI:** 10.3389/fonc.2025.1616883

**Published:** 2025-07-04

**Authors:** Rola Ghasoub, Basant Elsayed, Maria Benkhadra, Giuseppe Saglio, Jorge Cortes, Mohamed Elmarasi, Engy Elsayed, Ahmed Adel Elsabagh, Ibrahim Elmakaty, Abdelrahman Elsayed, Mohamed Yassin

**Affiliations:** ^1^ Department of Pharmacy, National Center for Cancer Care and Research Hamad Medical Corporation, Doha, Qatar; ^2^ Department of Medical Education, Hamad Medical Corporation, Doha, Qatar; ^3^ Department of Clinical and Biological Sciences, University of Turin, Turin, Italy; ^4^ Georgia Cancer Center, Augusta University, Augusta, GA, United States; ^5^ College of Medicine, Qatar University, Doha, Qatar; ^6^ Department of Hematology, Hamad Medical Corporation, Doha, Qatar

**Keywords:** obesity, tyrosine kinase inhibitors, chronic myeloid leukemia, weight loss pharmacotherapy, bariatric surgery, lifestyle modifications

## Abstract

**Background/Objectives:**

The rising prevalence of obesity presents significant challenges in managing chronic myeloid leukemia (CML). Bariatric surgery alters the bioavailability of oral medications, necessitating tailored treatment strategies for obese CML patients. This review aims to summarize the existing literature regarding CML management in patients with obesity and related surgeries, providing novel recommendations for their medical and surgical management.

**Methods:**

A narrative review was performed, analyzing English-language articles published up to 2024. Key aspects considered included the impact of obesity and surgery on the pharmacokinetics (PK) of tyrosine kinase inhibitors (TKIs), potential drug-drug interactions (DDIs) with weight loss medications, and the role of therapeutic drug monitoring (TDM). The review also examined the implications of obesity on CML patient outcomes and treatment milestones, aiming to establish a pathway for managing these patients.

**Results/Discussion:**

Clinical data on CML management in obese patients is limited. Gastric bypass surgery may impair TKI efficacy due to reduced gastric acid secretion, affecting drug absorption. Adjustments like deflating gastric bands may enhance TKI absorption. The concurrent use of proton pump inhibitors can significantly lower the efficacy of certain TKIs, while others can be safely co-administered. Notably, patients with prior bariatric surgery show delayed treatment response milestones and may require TKI dose adjustments based on molecular responses rather than TDM.

**Conclusion:**

Patients with CML planning bariatric surgery should receive comprehensive preoperative counseling regarding its effects on TKI therapy. Achieving deep remission pre-surgery is advisable, and delaying procedures when malabsorption is anticipated may enhance treatment outcomes. A multidisciplinary approach is essential in considering weight-loss medications, with further research needed to optimize TKI dosing in this population.

## Graphical Abstract

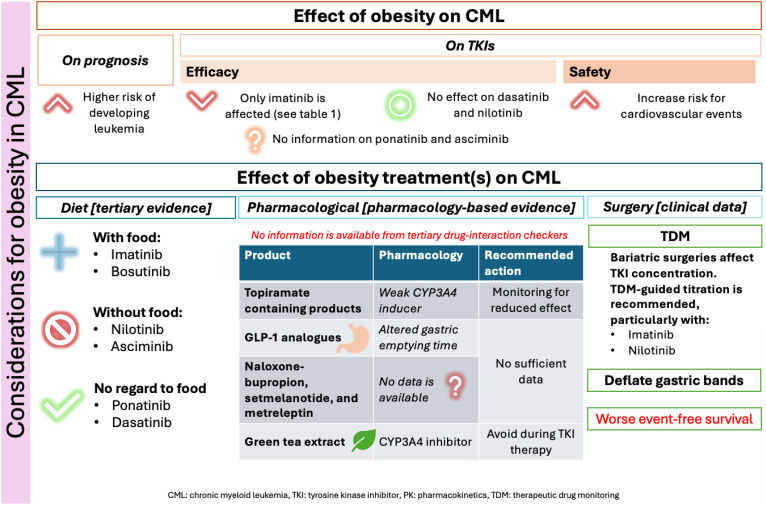

## Highlights

Obesity complicates the treatment of patients with chronic myeloid leukemia (CML), and bariatric surgery may impact how patients respond to their medications. This review examines the limited research on how obesity and weight loss surgeries affect the effectiveness of tyrosine kinase inhibitors (TKIs), which are commonly used to treat CML. The findings suggest that certain surgeries, like gastric bypass, could reduce drug absorption due to changes in gastric acidity, potentially making treatments less effective. Patients who have had these surgeries may need different dosing strategies for their TKIs and should avoid certain weight loss medications and herbs that can interfere with drug absorption. Careful planning and counseling are essential for patients with CML considering bariatric surgery to ensure they achieve deep remission before the procedure. Overall, a collaborative clinical approach is recommended for managing obesity in CML patients, while more research is needed on optimal dosing and treatment strategies.

## Introduction

Obesity is a chronic health condition that is increasingly prevalent across various age groups that affects one in eight individuals and contributes to approximately 300,000 deaths annually, making it the second leading cause of preventable mortality (1, 2). Furthermore, obesity in adulthood is significantly associated with increased risk of chronic myeloid leukemia (CML) and other hematological malignancies (3–6). Even after adjusting for various risk factors, obesity remained significantly linked to CML across all age groups (3, 7, 8). For instance, weight gain over 1 kg annually between ages 25 and 40 quadrupled the CML risk, possibly negating the effects of any preclinical weight loss (8).

**Figure 1:**
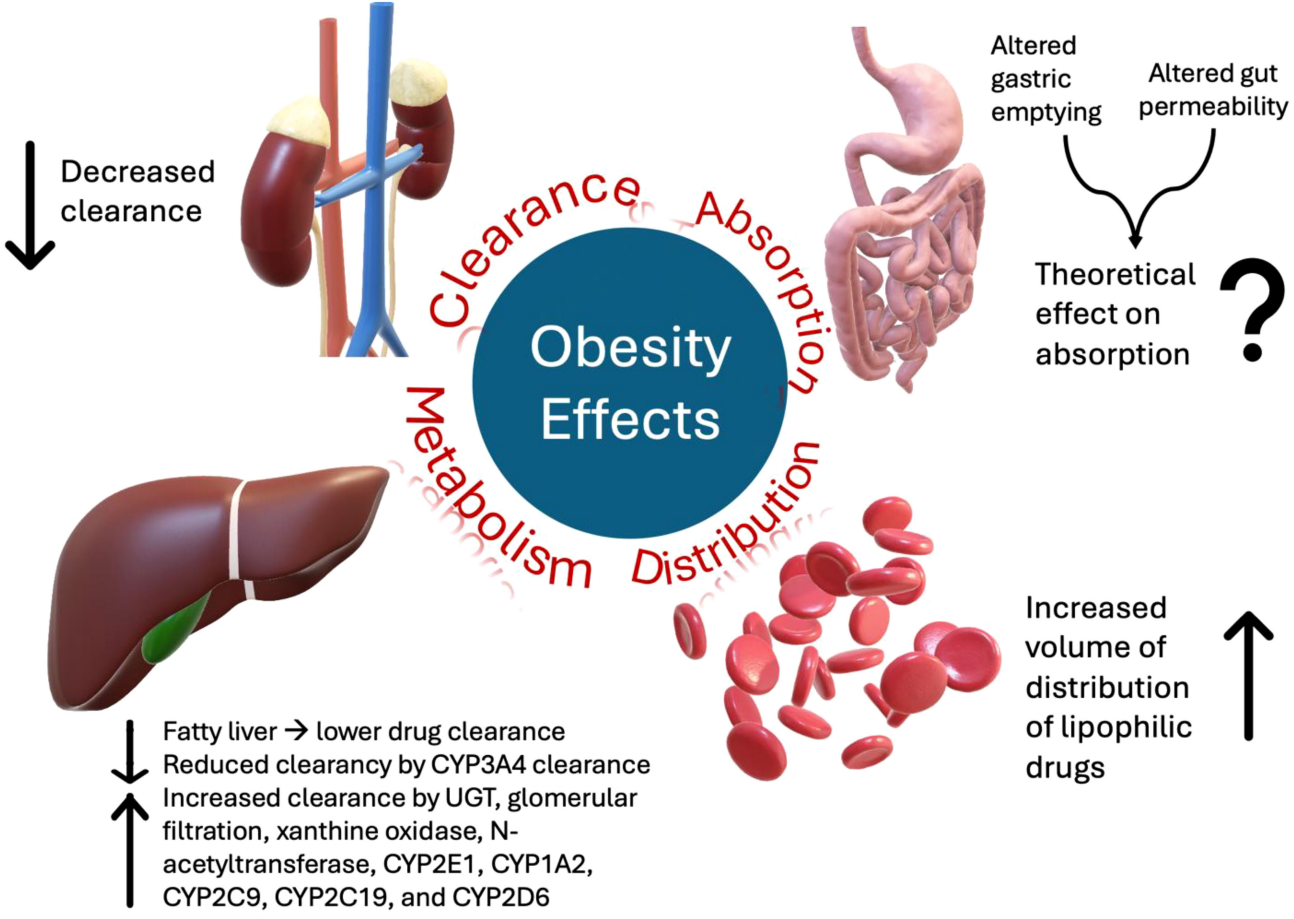
Summary of the effects of obesity on the pharmacokinetics of medications.

In addition, obesity can alter several physiological functions that influence the pharmacodynamics (PD) and pharmacokinetics (PK) of medications, as summarized in **Figure 1** (9). For instance, obesity may affect drug absorption by altering gastric emptying and gut permeability; however, oral bioavailability is not significantly affected in most cases (10). Moreover, the volume of distribution (Vd) correlates with the adipose body weight of lipophilic drugs. Therefore, a high percentage of adipose tissue in obesity is associated with a higher Vd of lipophilic drugs (9, 11).

Furthermore, obesity can alter drug clearance (CL) (9). In normal-weight patients, the typical ratio between lean, which typically increases CL, and adipose body weight was 4:1; however, in obesity, an increase in adipose tissue leads to an alteration of this ratio to 3:2, which decreases drug CL (9). Obesity can also impact various metabolic processes, including liver function, which is crucial for drug CL and metabolism. For example, obese patients have lower CL of drugs metabolized by cytochrome P450 (CYP) 3A4, which is the main metabolic pathway for all tyrosine kinase inhibitors (TKIs), i.e., the mainstay treatment for CML (11). On the other hand, obese patients tend to have higher CL and, hence, lower plasma concentrations of drugs metabolized via other metabolic pathways, such as uridine diphosphate glucuronosyltransferase (UGT), glomerular filtration, xanthine oxidase, N-acetyltransferase, CYP2E1, CYP1A2, CYP2C9, CYP2C19, and CYP2D6 (12). Obesity can also cause fatty changes in the liver, alter hepatic blood flow and reduce drug CL (13).

While the kidneys also play a significant role in drug elimination, the effects of obesity on these functions are unclear. Initially, obesity increases glomerular filtration rate (GFR) and renal drug CL due to higher cardiac output and renal perfusion (11). However, later in life, owing to persistently elevated intra-glomerular pressure and a strong correlation with chronic kidney disease (CKD), obesity is associated with a decline in renal drug CL (14, 15).

Given the differences in responses to treatment, dosage, and associated comorbidities, managing patients with obesity and bariatric surgeries has become a challenging task for CML patients. Several studies indicated that obesity can adversely affect treatment outcomes in CML patients by reducing the efficacy of TKIs (16). Furthermore, obesity-related comorbidities, such as cardiovascular diseases and diabetes mellitus, can complicate CML management and increase the risk of treatment-related adverse events (17). Consequently, healthcare providers are increasingly recognizing the importance of addressing weight management as an integral component of comprehensive care for CML patients. In addition, treatment modalities for obesity have varied from lifestyle changes to pharmacological therapies and weight-reducing surgical interventions. It remains unclear how the different modalities may affect the safety and efficacy of TKIs.

Current recommendations for CML treatment do not consider patient weight and body mass index (BMI) as factors when deciding which TKI doses are required for the patient (18). Furthermore, there is a scarcity of information on the management of patients with CML who have undergone obesity-related surgeries. This review aimed to scope and summarize the current evidence on the effects of obesity and obesity related surgeries in patients with CML and propose guidance for the management in this patient population.

## Methods

The review did not limit the research to a specific period, but rather aimed to provide a comprehensive, in-depth overview of the topic and to answer the following three specific clinical questions:

- How does obesity influence the response to therapy in patients with CML?
- How do obesity therapies (pharmacological and non-pharmacological) affect CML and the response to TKIs?
- Can therapeutic drug monitoring aid in TKI dosing in CML patients with obesity?

Studies included in the review were identified through keyword searches of PubMed, Scopus, Web of Science, Web of Conferences, and grey literature. Keywords searched included ‘obesity,’ ‘chronic myeloid leukemia,’ or ‘CML,’ ‘TKI therapy,’ ‘Tyrosine kinase inhibitors,’ and ‘CML therapy.’ In addition, tertiary evidence published on different TKIs and their PK, as well as manual searches of reference lists from primary articles found in the initial searches, were also conducted. Only research papers written in English were included. If the paper contained data relevant to answering our focused clinical questions, it was considered relevant and included in the review. B.E., M.Y., R.G., M.B., G.S., and J.C. were involved in conducting the research for this review.

## Results

**Figure 2:**
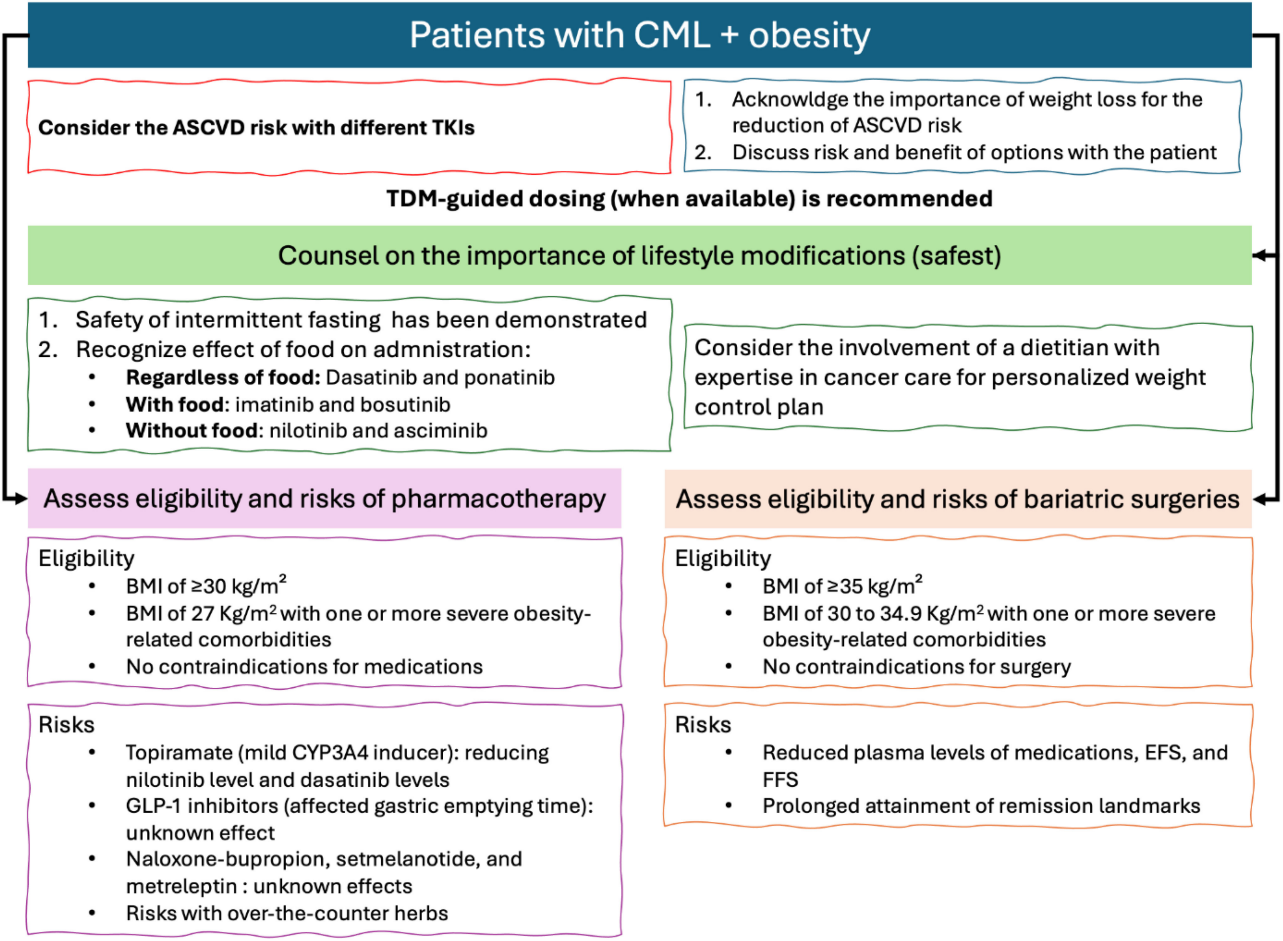
Summary of the recommendations on the management of CML with obesity.

**Figure 2** below summarizes the results and relevant recommendations for managing CML with obesity. Based on the current evidence, it is recommended to assess the Atherosclerotic Cardiovascular Disease (ASCVD) risk of patients before choosing the most appropriate TKI for CML treatment. This is because of the added combined risk factors for ASCVD with certain TKIs and obesity. Furthermore, according to all obesity management guidelines, lifestyle modifications are always recommended for obese patients (19). **Figure 2** emphasizes that lifestyle interventions are the safest obesity management strategies and recommended, with personalization of approaches, for all obesity patients (19). However, it is recommended to assess the effects of food on the absorption of TKIs, as summarized in **Figure 2** to ensure that the chosen diet plan does not affect their administration.

After establishing the importance of lifestyle interventions, as per the international guidelines for the management of obesity, patients should be assessed for their eligibility for pharmacotherapy or surgical interventions (eligibility is detailed in **Figure 2**) (20). Yet, each of those interventions may hold some risks for CML patients. These risks are summarized in **Figure 2**, based on the best available evidence.

### How can obesity affect the effectiveness of CML therapy?

#### Efficacy outcomes

Limited data is available regarding the exact changes in PK and PD of TKIs that are associated with obesity. As for the clinical effect, high BMI was only documented to affect the attainment of clinical landmarks with imatinib (21). In particular, high BMI in imatinib patients led to less achievement of major molecular remission (MMR) and delayed the time to attain MMR and complete cytogenetic response (CCyR), as shown in **Table 1** (21). In addition, a case reported (a 27-year-old patient with a BMI of 113 Kg/m^2^) by Yassin (2021), showed that initial imatinib doses in morbidly obese patients may achieve complete hematologic remission but require higher doses to achieve molecular remission (10). Neither dasatinib, nilotinib, nor bosutinib showed any clinically relevant associations when comparing patients with high versus normal BMI (22, 23). No data were found with ponatinib or asciminib.

**Table 1:** Effect of weight on the clinical effects of CML TKIs.

TKI	Effect of body weight
Imatinib	In comparison to patients with normal BMI, those with higher BMI (>25 Kg/m2) (21): - Required a significantly longer time to achieve CCyR - Had a significantly reduced rate of MMR - Required a significantly longer time to achieve MMR Increasing the dose of imatinib in morbidly obese patients, if tolerated, may assist in achieving molecular targets (10)
Dasatinib	No clinical effect was detected (22)
Nilotinib	No clinical effect was detected (22)
Bosutinib	No clinical effect was detected (23)
Ponatinib	No information found
Asciminib	No information found

#### Safety outcomes

Among the FDA-approved TKIs, nilotinib (n=795 reports), imatinib (n=130 reports), ponatinib (n=100 reports), and dasatinib (n=44 reports) had the highest ASCVD events reported in CML patients in the FDA Adverse Events Reporting System (FAERS) by June 30^th^, 2024 (24). Although FAERS is affected by the duration of post-marketing and reporting subjectivity, it still indicates the risk for ASCVD among the different TKIs (24). Prior guidance has been published on the assessment and management of cardiovascular risk with TKIs in CML, as well as potential drug interactions with cardiac medications (25). What is important to consider in patients with obesity is the significantly added risk that it carries for developing cardiovascular disease (26, 27). Therefore, the attainment of a healthy weight and adequate control of comorbidities in CML patients is recommended (25, 27). This is to potentially reduce the adverse effects of obesity on the safety outcomes for TKIs, particularly on ASCVD events.

### How can obesity-related therapies affect CML and response to TKIs?

#### Non-pharmacological therapies: lifestyle modifications

Weight management involves a comprehensive approach to achieving optimal weight. Recommendations should be tailored to individual needs, but general guidelines suggest a caloric deficit diet to reach daily 1200–1500 kcal for females and 1500–1800 kcal for males (19). This approach, often involving a progressive reduction in calorie intake, typically results in a weight loss of about 0.5 kg per week (19). Guidelines also advise on incorporating physical exercise into routines and provide extensive recommendations on types and durations (19). For patients with CML, lifestyle modifications are considered the safest weight loss approach when well tolerated, as they do not appear to affect the efficacy of TKI therapy. However, it is important to consider how diet practices may affect TKI PK.

#### Effects of food on TKI therapy

Instituting caloric deficits and weight reduction diets are important aspect of obesity treatment. A popular diet-modification strategy is intermittent fasting (28). The safety of TKI administration in CML patients who practice intermittent fasting was demonstrated in a 3-year retrospective observational study (29). However, patients should be counseled on the appropriate administration of TKIs with regard to their diet. **Table 2** below demonstrates how meals can affect the PK of TKIs in CML.

**Table 2:** Effect of food on the exposure to CML TKIs and labelling administration recommendations with regard to meals.

TKI	Effect of food
Imatinib	High-fat meals can minimally decrease the exposure to imatinib (by ˜ 7%) (30). Therefore, it is still recommended to administer imatinib after food to reduce GI upset (31), which exceeds the reduction in exposure.
Dasatinib	Meals can increase the exposure to dasatinib by 14 to 21% (30). Therefore, the manufacturer recommends administering it without regard to meals (30).
Nilotinib	Food can significantly increase exposure to nilotinib [low fat: by 15 to 29%; high fat: 43 to 82%] (30). Therefore, the manufacturer recommends administering nilotinib on an empty stomach (30).
Bosutinib	High-fat meals can increase the exposure to bosutinib by 54 to 70% (30). Therefore, it is recommended to take bosutinib with food (30).
Ponatinib	Food does not affect exposure to ponatinib (30). Therefore, it is recommended to take ponatinib without regard to meals (30).
Asciminib	The exposure to asciminib is decreased by 30 to 64% by low and high fat meals, respectively (32). Therefore, the manufacturer recommends administering asciminib on empty stomach (33).

### Non-pharmacological therapies: weight loss surgeries

Weight loss surgeries are effective methods to achieve long-term weight control (20). The most common weight loss surgeries are the gastric sleeve and bypass surgeries (34). These strategies for weight control are usually reserved, as per the latest guideline updates, for patients with a BMI of ≥ 35 kg/m² or in those with a BMI of 30 to 34.9 Kg/m^2^ with one or more severe obesity-related comorbidities, such as diabetes (20).

### General effects of bariatric surgeries on the pharmacokinetics of medications

Given the anatomical and physiological changes that occur in the gastrointestinal tract, it may be anticipated that the PK of oral medications may be affected. This is because bariatric surgeries can affect the available surface area for absorption, alter pH, and affect gastric volume and motility, depending on the type of surgeries performed (35). Bariatric surgeries can also alter glucagon-like-peptide-1 (GLP-1) and other gastric hormone levels (36). This leads to alterations in gastric emptying time and affects the absorption of medications (36). In addition, following bariatric surgeries, liver size has been observed to decrease, which leads to reduced activity of CYP enzymes that are essential for first-pass metabolism (35). This can also happen in surgeries bypassing portions of the small intestine where considerable concentrations of CYP P450 enzymes exist (36). Further literature scoping was performed to explore if those effects reflected on clinical outcomes among CML patients receiving TKIs who also underwent bariatric surgeries.

### Treatment of CML in patients with obesity-related surgeries

Pavlovsky (2009) reported a case of a 36-year-old, morbidly obese CML patient (130 Kg, BMI not reported), who received imatinib 400 mg/day and achieved a complete molecular response (37). She underwent sleeve gastrectomy four years after imatinib initiation (37). The patient’s preoperative trough plasma concentration (1558 ng/ml) was consistent with the target imatinib levels reported in the literature (>/= 1000 ng/ml) (37). However, her trough concentrations after surgery were 46-60% lower (629-836 ng/ml) than the preoperative value (37). Despite this, the patient remained in complete molecular remission for one year after surgery (37).

Later on, Liu (2011) reported a case of a 54-year-old morbidly obese CML patient (BMI 50 Kg/m^2^) who was being treated with imatinib 400 mg/day and achieved CCyR after the dose was increased to 400 mg twice daily (38). She was briefly switched to dasatinib 100 mg/day due to gastrointestinal adverse events with the higher dose of imatinib; however, she still experienced fluid retention adverse events with dasatinib (38). As per the authors, her BCR::ABL1 level was approaching MMR, so she was switched back to imatinib 400 mg/day, on which she achieved a 24-hour trough imatinib plasma level of 965 ng/mL (38). After that, the patient underwent laparoscopic biliopancreatic diversion with duodenal switch (BPD/DS), as well as cholecystectomy and appendectomy (38). At 22 months post-BPD/DS, the patient’s BCR::ABL1 increased by 0.5 logs, but she maintained CCyR (38). Her plasma levels of imatinib at the time had dropped by 83% to 166 ng/mL (38). The authors then increased the dose of imatinib to 400 mg twice daily, leading to a steady increase in plasma levels as her weight stabilized over the following 6 months (540 ng/mL at 3 months [72 Kg] and 2,124 ng/mL at 6 months [65 Kg]) (38). Unfortunately, her bone marrow showed 5% Philadelphia-chromosome-positive cells, and she was rechallenged with dasatinib 100 mg/day (38).

Furthermore, a 67-year-old CML patient with a medical history significant for hypertension, hypothyroidism and Roux-enY laparoscopic gastric bypass (RYGB) 12 years prior to her CML diagnosis (39). She was started on imatinib 400 mg/day and achieved MMR at 6 months (39). However, the patient’s quality of life was debilitated by multiple imatinib-related adverse events (grade 2 myalgia, grade 2 nausea, grade 1 pruritus, and bilateral lower limb grade 2 blisters) (39). Therefore, she was switched to nilotinib 400 mg BID, following which all adverse events were resolved, and MMR was maintained (39).

The cases reported by Yassin (2021) reported better results with imatinib post-bariatric surgeries and highlighted the importance of gastric band deflation for TKI absorption (10, 40). On the other hand, Lau reported relatively durable remission (lasting a few years) with second-generation TKIs, particularly nilotinib, ponatinib, and bosutinib (16). They also observed time-dependent improvement in imatinib levels following bariatric surgery (16), similar to the observation made by Liu (2011) (38).

Furthermore, a case-control study by Haddad (2023), which spanned 19 years, showed a significantly slower time to achieving early molecular (3‐month BCR::ABL1 < 10% International Scale), CCyR, and MMR in CML patients who had bariatric surgeries compared to those who didn’t (41). It also showed that compared to the control groups, patients who underwent bariatric surgeries had significantly inferior event-free and failure-free survival rates (41). As for differences between TKIs, there was a non-statistically significant tendency for patients on second-generation TKIs to obtain faster responses (41).

### Pharmacological therapies: weight loss medications

Pharmacotherapy for obesity is recommended for patients with a BMI >30 kg/m² or >27 kg/m² accompanied by obesity-related complications (42). However, these pharmacotherapies should only be utilized in combination with lifestyle modifications (42). Recent obesity management guidelines have recommended multiple pharmacotherapy options, depending on individual risk factors, including orlistat, GLP-1 analogues [liraglutide or semaglutide or tirzepatide], naltrexone-bupropion, phentiramine-topiramate, setmelanotide, and metreleptin (42).

The first concern regarding the use of weight loss medications in CML patients is drug-drug interactions (DDIs). Upon searching tertiary resources, no clinically significant drug interactions were noted, although topiramate-containing products are known to be mild inducers of CYP3A4 and both dasatinib and nilotinib are substrates of this enzyme (43–45). This indicates that metabolism-based DDIs are not concerning between FDA-approved obesity and CML-approved pharmacotherapies.

While many of the new weight loss medications do not affect metabolizing enzymes, some (GLP-1 analogues) are known to affect gastric conditions, particularly gastric emptying time, which in turn can affect the absorption of oral medications (42). Whether this can affect the clinical outcomes of TKIs in CML is yet to be unraveled clinically. Moreover, the possible effects of naloxone-bupropion, setmelanotide, and metreleptin have any potential DDIs with TKIs are yet to hypothesized. Lastly, it is important to counsel patients regarding the drug interactions with complementary medicine products that are frequently purchased over the counter for weight loss. For example, green tea extracts can inhibit CYP3A4 enzymes, which may affect the levels of medications that are degraded by it (46).

### Can therapeutic drug monitoring aid in TKI dosing in CML?

Maroselli (2025) has reported their experience with 60 CML patients among whom BMI (both underweight and overweight ranges) and age were the only factors associated with imatinib trough levels (47). TDM-guided dosing improved the attainment of target imatinib levels and personalized the dose according to what each patient needs (47). Furthermore, the results presented in **Table 3** showed that TKI plasma levels were affected by body weight and bariatric surgeries, this raises the hypothesis that with TDM-guided dosing, achieving target plasma levels may be possible.

**Table 3:** Summary of the clinical evidence on the effects of bariatric surgeries on TKI effect.

Study	Number of patients		TKI	Surgery type	Conclusion
Pavlovsky (37) (2009) Case report	1		Imatinib 400 mg/day	Sleeve gastrectomy post-TKI	Sleeve gastrectomy causes a reduction in imatinib plasma drug levels, although efficacy was not affected.
Liu (38) (2011) Case report	1		Imatinib 400 mg/day	BPD/DS post TKI	BPD/DS led to 83% reduction in imatinib plasma levels with maintenance of CCyR but loss of MMR. Doubling the dose improved plasma levels as the weight stabilized.
Centrone (39) (2020) Case report	1		Imatinib 400 mg/day	RYGB before TKI	Patient achieved MMR at 6 months but had significant imatinib toxicities requiring treatment modification.
			Nilotinib 400 mg twice/day		Adverse events from prior therapy resolved with nilotinib and MMR was maintained.
Yassin (10) (2021) Case series	5 patients	Case 2*^§^ (40)	Dasatinib 100 mg/day	Sleeve gastrectomy after TKI	Lost hematologic response ➔ switched to imatinib.
			Imatinib 400 mg/day		Achieved CHR, CCyR, and MMR (10)
		Case 3^§^ (40)	Nilotinib 300 mg twice/day	Sleeve gastrectomy before TKI	Patient had complete hematological response but not cytogenetic ➔ switched to imatinib.
			Imatinib		Achieved CHR, CCyR, and MMR (10) *Imatinib dose was not* sp*ecified*
		Case 4	Imatinib 400 mg/day	Gastric band before TKI	Before band deflation, patient only achieved CHR. After deflation, patient achieved molecular target.
		Case 5	Nilotinib	Sleeve gastrectomy before TKI	Patient had complete hematological response but not MMR ➔ switched to imatinib. *Nilotinib dose was not* sp*ecified*
			Imatinib		2 log reductions but not MMR ➔ switched to pegylated interferon ➔ progression to blast crisis. *Imatinib dose was not* sp*ecified.*
Haddad (41) (2023) Case-control	(22 cases Vs. 44 controls)		Imatinib (8 cases)	Gastric sleeve (11 cases)	Bariatric surgery patients had inferior event-free survival and failure-survival than the control group. The rate of achieving early molecular remission was also slower in bariatric patients. *TKI doses were not* sp*ecified.*
						Dasatinib (10 cases)
			Sleeve gastrectomy (7 cases)			
				Nilotinib (3 cases)	
			Gastric banding (4 cases)		
				Bosutinib (1 case)	
			Lau (16) (2024) Case series	4 patients		Case 1	Imatinib 400 mg/day	RYGB before TKI	Intolerant; switched to nilotinib.
Nilotinib 400 to 600 mg twice/day	400 mg twice/day: Achieved MMR but had adverse events ➔ dose reduced. 300 mg twice/day: subtherapeutic levels + adverse events ➔ switched to dasatinib. Rechallenge at 300 mg to 600 mg twice/day: Persistent lack response, subtherapeutic levels, and adverse events ➔ switched to ponatinib.							
Dasatinib 50 to 180 mg/day	100 mg/day: adverse events ➔ dose reduced to 70 mg/day then 50 mg/day 50 mg/day: initially maintained MMR for 7 months ➔ PPI stopped, and dose increased. 140 mg to 180 mg/day: Persistent lack response despite therapeutic levels ➔ rechallenged with nilotinib							
Ponatinib 15 to 45 mg/day	45 mg/day: adverse events ➔ dose reduced to 30 mg/day. 30 mg/day: MMR reached, and adverse events reduced ➔ maintenance dose. 15 mg/day: MMR maintained.							
Case 2	Nilotinib 150 to 450 mg twice/day	Gastric banding following TKI			150 to 450 mg twice/day: MMR initially maintained but required multiple interruptions and dose adjustments due to adverse events. After 2 years and 10 months, MMR was lost ➔ switched to bosutinib.
	Bosutinib 100 to 400 mg/day				400 mg/day (post ramp-up): severe headache requiring dose reduction to 200/day then 100 mg/day ➔ rechallenged with 400 mg/day. 400 mg/day: MMR achieved, maintained and tolerated at last follow-up (3 years)
Case 3	Bosutinib 400 mg/day	RYGB before TKI			Subtherapeutic level with rising BCR::ABL1 at 9 months.
Case 4	Imatinib 400 mg/day	RYGB after TKI			Initially subtherapeutic levels that improved after 16 months without affecting molecular response.

*First case did not include bariatric surgery, ^§^some details were also reported in the abstract publication as cited. TKI, tyrosine kinase inhibitor; BPD/DS, biliopancreatic diversion with duodenal switch; CCyR, complete cytogenetic remission; RYGB, Roux-enY laparoscopic gastric bypass; MMR, major molecular remission; PPI, proton pump inhibitor.

## Discussion

This is the first review to provide a comprehensive summary of the literature surrounding the pharmacological and surgical considerations of obesity in patients with CML. As illustrated in this review, there is a need to fill several gaps in the literature, starting with real-world clinical data and practical applications. With lifetime treatment, the majority of CML patients have achieved complete and lasting cytogenetic responses owing to the remarkable effects of TKIs on patient management. This significant shift in CML prognosis presents new clinical and scientific challenges that must be addressed. Currently, bariatric surgery is widely used, and evidence suggests a significant reduction in obesity-associated mortality (48).

It has been demonstrated that the changes resulting from these procedures can affect the bioavailability of oral drugs (49, 50). Although gastric bypass remains the most effective and long-lasting weight-loss option for individuals with severe obesity, there is a need to create customized treatment approaches tailored specifically for these patients. Based on the reported cases in the literature, gastric bypass surgery may influence clinical response to TKI therapy; hence, it is not recommended for individuals with severe obesity (38–40). In clinical practice, this underscores the need for clinicians to evaluate the dosing strategy for CML patients post-surgery actively. Adjustments such as increasing the TKI dosage may be necessary, but this must be balanced against the risk of toxicity (16, 41, 42).

Moreover, identifying the appropriate timing for TKI therapy initiation post-surgery is essential to avoid unnecessary delays, which could compromise patient outcomes. The use of TDM in obese patients becomes increasingly relevant here, as it can help clinicians optimize dosing based on the patient’s molecular response rather than relying solely on traditional PK parameters (47).

While TDM has historically been explored, particularly for imatinib, its role in routine clinical practice remains controversial. Early studies suggested a potential association between imatinib trough levels and treatment outcomes, with some recommending target trough levels ≥1000 ng/mL to optimize response (51, 52). However, subsequent investigations, failed to consistently demonstrate a direct correlation between plasma levels and treatment efficacy, particularly for second-generation TKIs (53, 54). Consequently, TDM has not been widely adopted and is not currently recommended as standard of care in most clinical settings. In our review, we highlight case-based and observational data where TDM-guided dosing was explored, particularly post-bariatric surgery. Nevertheless, these reports remain limited in number and quality. Hence, further prospective studies are warranted to clarify the clinical utility, standardization, and cost-effectiveness of TDM in optimizing TKI therapy among complex populations such as patients with obesity or gastrointestinal alterations.

In addition to the decreased absorption of TKIs in patients who have undergone bariatric surgery, other factors, such as the simultaneous use of proton pump inhibitors or the presence of comorbidities, may impact the attainment of an optimal response in patients with CML (55, 56). Therefore, it is paramount for clinicians to engage in comprehensive medication reconciliation and patient education to mitigate these risks.

The limitations of this review must be acknowledged. First, there is a lack of available data on the interactions between several weight loss medications and TKIs, such as orlistat and GLP-1 agonists. In addition, only case reports are available on the effect of bariatric surgery on the PK of TKIs, which is insufficient for drawing definitive conclusions. Furthermore, there is limited data on the delayed effects of bariatric surgery on physiological changes and how they affect drug PK in the long term. Overall, with the current scarcity of literature and the level of evidence, there is room for future research to address this gap. A multidisciplinary team, including a bariatric surgeon, CML specialist, dietitian, and clinical pharmacist, is essential for shared decision-making with the patient.

## Conclusions

Based on our review, we recommend that postoperative patients to be closely monitored for clinical response and medication levels in the blood (if feasible); and further dose adjustment or drug switching should not be delayed if indicated. However, the lack of readily available assays for measuring TKI levels in clinical settings limits the viability of this approach. Therefore, alternative strategies include escalating TKI dosage in cases of response failure or warning signs observed during milestone monitoring. Lastly, when the decision to undergo bariatric surgery occurs post-CML diagnosis, it is advisable to enhance the response to achieve deep remission before surgery. Moreover, there may be merit in postponing the surgery to allow for a safety margin in the CML response if significant malabsorption is anticipated because of the procedure.
